# Supplementary material for: Acute Macular Neuroretinopathy after SARS-CoV-2 Infection: An Analysis of Clinical and Multimodal Imaging Characteristics
Source: Diagnostics (Basel). 2023 Dec 5;13(24):3600. doi: 10.3390/diagnostics13243600 (PMC10742651; doi:10.3390/diagnostics13243600)
Supplement: Supplementary file 1 [file diagnostics-13-03600-s001.zip › diagnostics-2631153-supplementary.pdf]

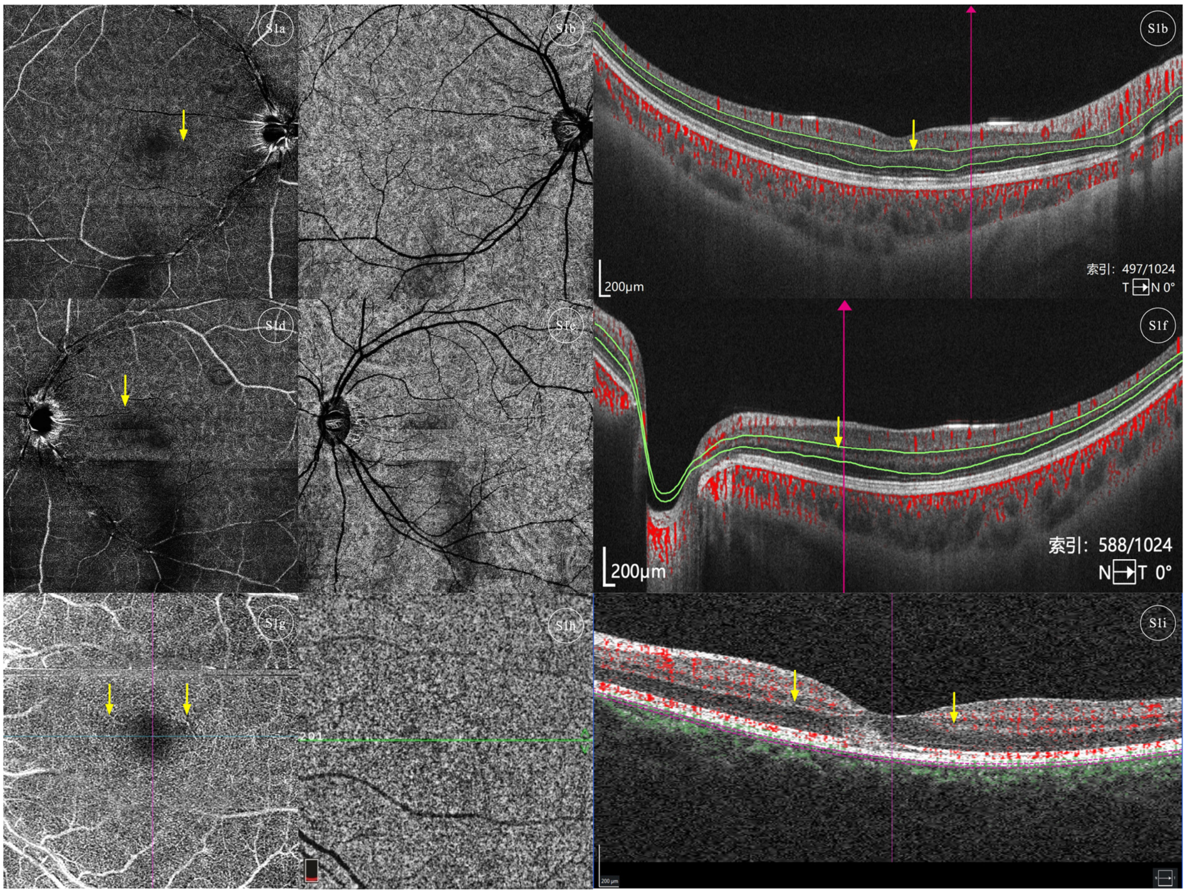

Supplementary Figure S1. En face OCTA images with corresponding OCT B-scans with flow overlay of cases 1 and 2. S1a and S1b show DCP and choriocapillaris layer of right eye of case 1, respectively. S1c shows corresponding OCT B-scan of the same eye with flow overlay and flow attenuation at DCP layer (yellow arrows). S1d and S1e show DCP and choriocapillaris layer of left eye of case 1, respectively. S1f shows corresponding OCT B-scan of the same eye with flow overlay and flow attenuation at DCP layer (yellow arrows).

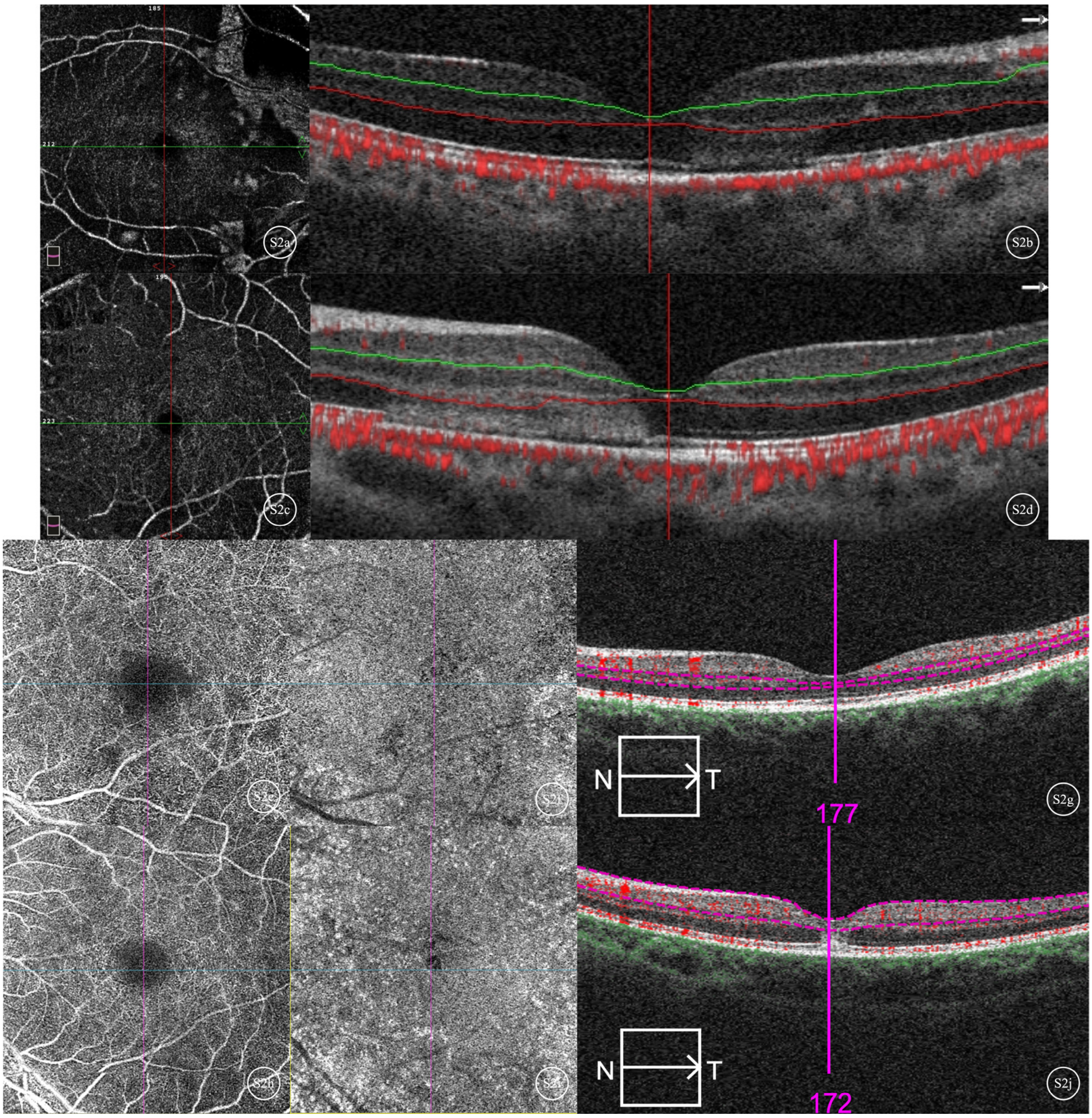

Supplementary Figure S2. En face OCTA images with corresponding OCT B-scans with flow overlay of cases 5, 6 and 7. S2a and S2c show DCP layers of right and left eyes of case 6, respectively. S2b and S2d show corresponding OCT B-scan of the same eye with flow overlay of right and left eyes of case 5, respectively. The poor quality of the en face OCTA images caused by artifacts prevented meaningful interpretation of the DCP perfusion in the affected eyes. S2e and S2f show DCP and choriocapillaris layer of left eye of case 6, respectively. S2g shows corresponding OCT B-scan of the same eye with flow overlay. S2h and S2i show DCP and choriocapillaris layer of left eye of case 7, respectively. S2j shows corresponding OCT B-scan of the same eye with flow overlay. It could not be categorically ruled out that areas of flow signal attenuation observed on choriocapillaris layer (S2f and S2i) were caused by projection artifacts.

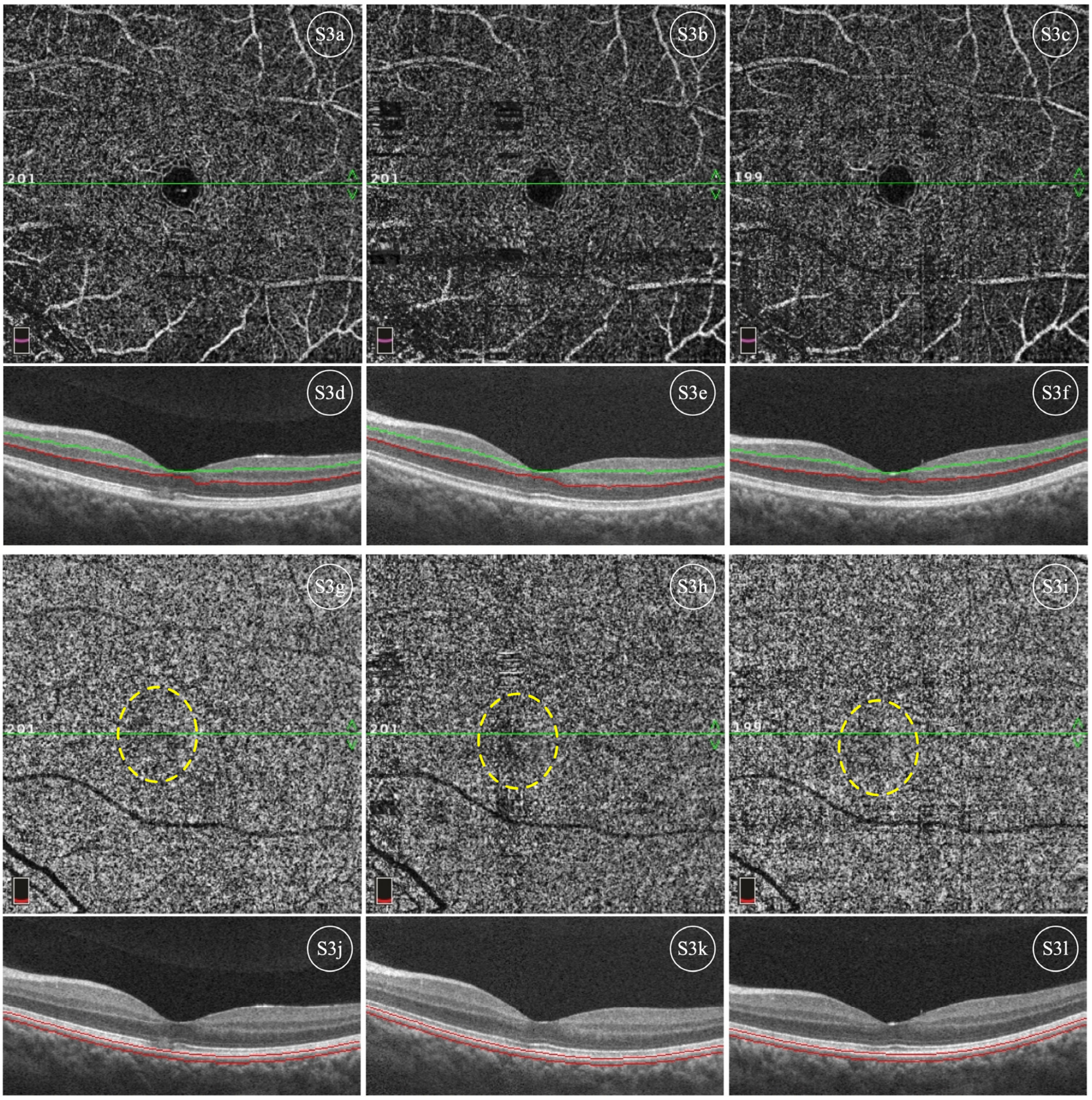

Supplementary Figure S3. Follow-up en face OCTA images with corresponding OCT B-scans of case 2. S3a, S3b and S3c show en face DCP images of left eye of case 2 at 2-, 26- and 46-day follow-ups. S3d, S3e and S3f show corresponding OCT B-scans of left eye of case 2 at 2-, 26- and 46-day follow-ups. S3g, S3h and S3i show en face choriocapillaris images of left eye of case 2 at 2-, 26- and 46-day follow-ups. S3j, S3k and S3l show corresponding OCT B-scans of left eye of case 2 at 2-, 26- and 46-day follow-ups. Encircled on S3g, S3h and S3i are areas of flow signal attenuation on choriocapillaris layer, which persisted even after resolution of focal ONL hyperreflectivity.
